# ROCK Is Involved in Vasculogenic Mimicry Formation in Hepatocellular Carcinoma Cell Line

**DOI:** 10.1371/journal.pone.0107661

**Published:** 2014-09-19

**Authors:** Ji-Gang Zhang, Xiao-Yu Li, Yu-Zhu Wang, Qi-Di Zhang, Sheng-Ying Gu, Xin Wu, Guan-Hua Zhu, Qin Li, Gao-Lin Liu

**Affiliations:** 1 Department of Pharmacy, Shanghai First People's Hospital, School of medicine, Shanghai Jiao Tong University, Shanghai, P.R. China; 2 Department of Gastroenterology, Shanghai First People's Hospital, School of medicine, Shanghai Jiao Tong University, Shanghai, P.R. China; Northwestern University, United States of America

## Abstract

Ras homolog family member A (RhoA) and Rho-associated coiled coil-containing protein kinases 1 and 2 (ROCK1 and 2) are key regulators of focal adhesion, actomyosin contraction and cell motility. RhoA/ROCK signaling has emerged as an attractive target for the development of new cancer therapeutics. Whether RhoA/ROCK is involved in regulating the formation of tumor cell vasculogenic mimicry (VM) is largely unknown. To confirm this hypothesis, we performed *in vitro* experiments using hepatocellular carcinoma (HCC) cell lines. Firstly, we demonstrated that HCC cells with higher active RhoA/ROCK expression were prone to form VM channels, as compared with RhoA/ROCK low-expressing cells. Furthermore, Y27632 (a specific inhibitor of ROCK) rather than exoenzyme C3 (a specific inhibitor of RhoA) effectively inhibited the formation of tubular network structures in a dose-dependent manner. To elucidate the possible mechanism of ROCK on VM formation, real-time qPCR, western blot and immunofluorescence were used to detect changes of the key VM-related factors, including VE-cadherin, erythropoietin-producing hepatocellular carcinoma-A2 (EphA2), phosphoinositide 3-kinase (PI3K), matrix metalloproteinase (MMP)14, MMP2, MMP9 and laminin 5γ2-chain (LAMC2), and epithelial-mesenchymal-transition (EMT) markers: E-cadherin and Vimentin. The results showed that all the expression profiles were attenuated by blockage of ROCK. In addition, *in vitro* cell migration and invasion assays showed that Y27632 inhibited the migration and invasion capacity of HCC cell lines in a dose-dependent manner markedly. These data indicate that ROCK is an important mediator in the formation of tumor cell VM, and suggest that ROCK inhibition may prove useful in the treatment of VM in HCC.

## Introduction

Vasculogenic mimicry (VM) was first described by Maniotis et al. [1] as a new blood supply system independent of endothelial vessels in malignant melanoma. It reflects the plasticity of aggressive tumor cells that express vascular cell markers and line tumor vasculature as has been demonstrated in many malignant tumors including HCC, the third most common cause of cancer mortality worldwide [2]–[5]. As extensive signaling pathways involved in the pathogenesis of HCC reflect the heterogeneity and complexity of liver carcinogenesis, understanding the roles of these pathways in the pathogenesis of HCC is essential for the effective prevention and treatment of HCC. Recently, a growing number of *in vitro* and *in vivo* studies using tumor-derived cell lines, primary tumors and animal cancer models strongly suggest that altered Rho GTPase signaling plays an important role in the development and progression of HCC. The overexpression or repression of GTPases or some upstream or downstream elements of Rho signaling has been reported to be associated with many human tumors [6].

The presence of VM was reported to be associated with a high tumor grade, short survival, and invasion and metastasis [7], [8]. Metastasis is the biggest threat to survival of patients with solid tumors, and cell migration is a pivotal step in metastasis [9]. Indeed, loss of polarity, disruption of cell-cell contacts and increase in cell motility are key events in the acquisition of the pro-invasive and metastatic phenotypes [10]. Interestingly, the RhoA small GTPase and its serine/threonine kinase downstream effector (ROCK1 and 2) control a wide variety of ubiquitous biological processes including the acquisition of unlimited proliferation potential, survival and evasion from apoptosis, tissue invasion differentiation, gene expression, and in particular, regulation of cell detachment, cell movement and establishment of metastasis [6], [11]. In addition, VM formation involving tumor cells mimics endothelial cells consisting of a type of mesenchymal cells, which is similar to epithelial to EMT, a process whereby fully differentiated epithelial cells lose epithelial characteristics and acquire mesenchymal ones. EMT is proposed to be a crucial mechanism regulating the initial steps in metastatic progression [12]. It has been demonstrated that Rho activity is required to induce EMT in a number of cell typesm [13], [14]. However, RhoA/ROCK as a therapeutic target for VM has not been documented.

Since the introduction of VM, a plethora of studies have attributed mechanistic insights into the induction, formation and targeting of VM across a variety of cancers. However, the pathogenesis of VM is a complex process involving extensive signaling pathways. The first two proteins identified to play a role in mediating melanoma VM are VE-cadherin, a cell-cell adhesion molecule associated with endothelial cells, and EphA2, an epithelial cell associated kinase involved in ephrin-A1-induced angiogenesis [15]. VE-cadherin regulates EphA2 activity by mediating its ability to get phosphorylated through interactions with its membrane-bound ligand ephrin-A1, thus activating the expression of PI3K, MMP14 and MMP2. Both MMP14 and MMP2 promote the cleavage of LAMC2 into promigratory γ2′ and γ2× fragments, which in turn stimulates migration, invasion, and VM in melanoma cells [16]. The MMPs family, especially MMP2, MMP9 and MMP14, together with LAMC2, PI3K, VE-cadherin and EphA2, participate in the key signaling pathway of VM formation [17]. Interestingly, Rho GTPases can also regulate the production of MMPs, affecting matrix remodeling and tumor cell invasion. Rho can induce the expression or secretion of MMPs [18]. The purpose of this study was to determine whether RhoA/ROCK signaling was involved in HCC cell VM formation *in vitro*, and explore the possible mechanism.

In this study, we sought to characterize the ability of RhoA/ROCK signaling to act as a mediator in the process of VM formation. It was found that the expression of RhoA, ROCK1 and ROCK2 proteins varied in HCC cell lines, indicating different VM formation potentials. Furthermore, we examined the ROCK paralog-specific roles in EMT to investigate the possible mechanism of its contribution to VM formation. Finally, we blocked the RhoA/ROCK pathway with the pharmacological inhibitor exoenzyme C3 and Y27632 and analyzed the biological effects using *in vitro* angiogenic assays to monitor tube formation, the key VM-related factors expression, migration and survival.

## Results

### Correlations between the expression level of RhoA/ROCK and the capacity of VM formation

**Figure 1:**
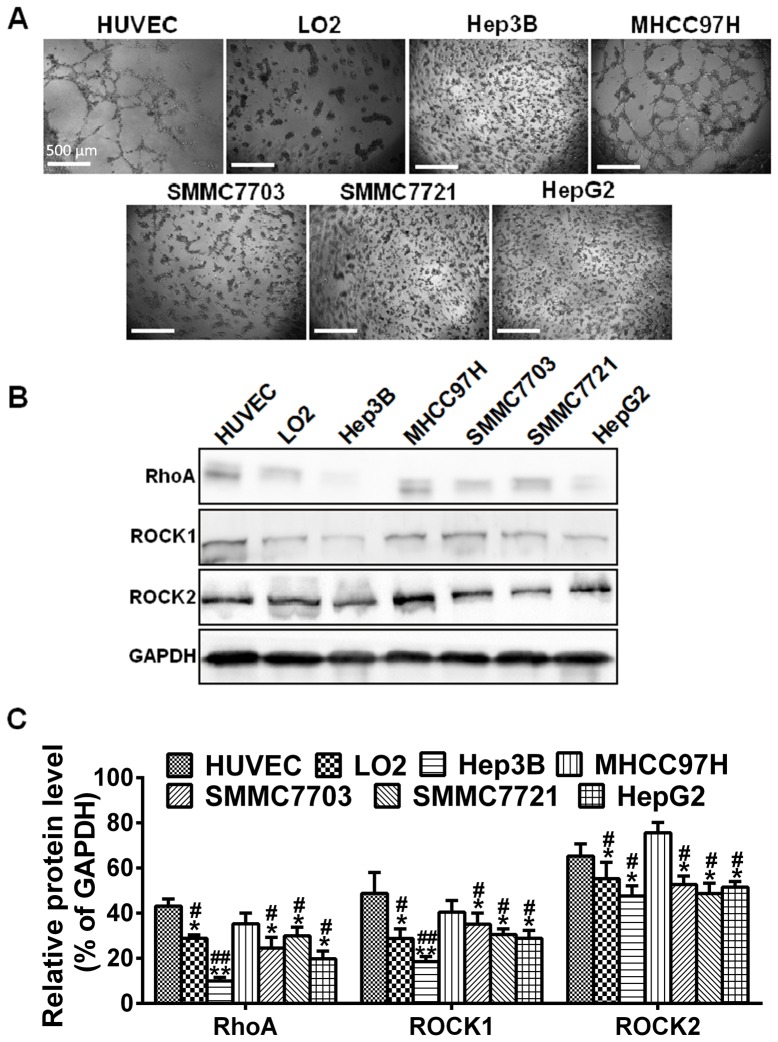
VM capacity and expression of RhoA, ROCK1 and ROCK2 in HCC cell lines. (**A**) Formation of tubular network structures on Matrigel by HUVEC and LO2 compared to HCC cell lines. Cells were seeded into Matrigel-coated 24-well plates and incubated at 37°C. Photographs were taken after 24 h, with original magnification of 40×, scale bars represent 500 µm. (**B**) Western blot was performed to confirm the protein expression of RhoA, ROCK1 and ROCK2 in HCC cell lines and LO2, using HUVEC as a positive control. (**C**) Relative densities (RD) are presented as mean ± S.E. (n = 3) of the fold change relative to the internal control. GAPDH was used as an internal control for protein loading. *P<0.05 and **P<0.01 *vs.* HUVEC and **^#^** *P*<0.05 and ***^##^*** *P*<0.01 *vs.* MHCC97H.

To examine the involvement of RhoA/ROCK signaling in VM formation, VM capacity and expression of RhoA, ROCK1 and ROCK2 were investigated in five HCC cell lines with different metastasis potentials and one hepatocyte cell line. Human umbilical vein endothelial cells (HUVEC) were used as positive controls. Test of formation of tubular network structures on Matrigel was used to determine the capacity of VM *in vitro*. It was found that MHCC97H and HUVEC had the capacity of VM formation (Figure. 1A). However, the other HCC cell lines failed to form any tube or network. As shown in (Figure. 1B), both RhoA and ROCK were present at detectable levels. Statistical analysis showed that the expression level of RhoA, ROCK1 and ROCK2 in HUVEC and MHCC97H was significantly higher than that in other HCC cell lines (Figure. 1C, *P<0.05 and **P<0.01 *vs.* HUVEC and **^#^** *P*<0.05 and ***^##^*** *P*<0.01 *vs.* MHCC97H). However, there were no statistically significant differences between MHCC97H and HUVEC (*P*>0.05 *vs.* HUVEC). These data indicate that compared with the low-expression cells, HCC cells with higher active RhoA/ROCK expression were more likely to form VM channels, suggesting that the RhoA/ROCK signaling pathway was positively correlated with the potential of VM formation in HCC cells.

### Effect of RhoA and ROCK inhibition on MHCC97H cell VM formation

**Figure 2:**
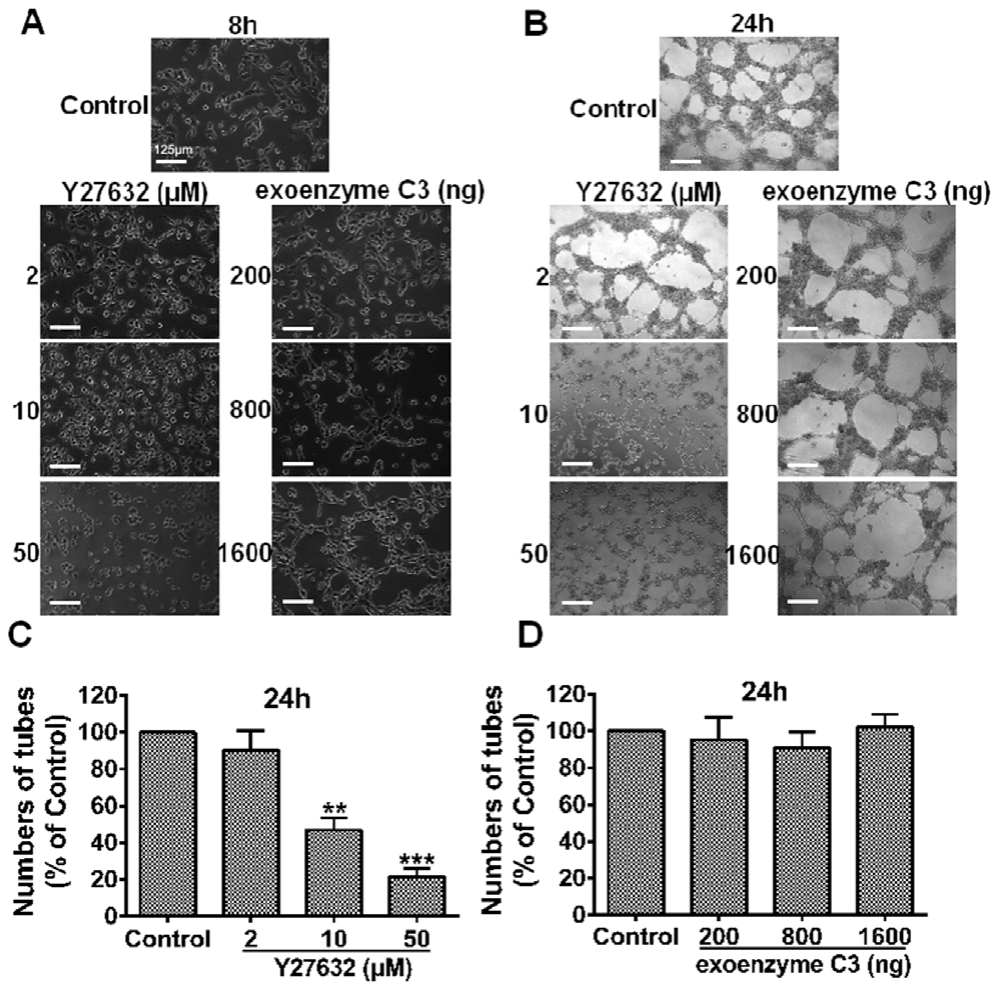
The impact of increased doses of Y27632 or exoenzyme C3 on VM formation. VM formation of MHCC97H cells cultured in Matrigel-coated 24-well plates, with culture medium containing Y27632 (2, 10 or 50 µM) or exoenzyme C3 (200, 800 or 1600 ng) for 8 h (**A**) or 24 h. (**B**). Photographs were taken at the indicated time points, and original magnification was 100×, scale bars represent 125 µm. (**C**), (**D**) Quantitative analysis of the mean number of tube-like structures formed from six randomly chosen areas in 3D cultures. Data are expressed as mean ± S.E. from three independent experiments, with significant differences from control designated as **P<0.01 and ***P<0.001.

Exoenzyme C3 and Y27632 were chosen as RhoA and ROCK inhibitors, respectively. To see whether blockage of RhoA/ROCK exerted an inhibitory effect on MHCC97H cell-mediated neovascularization *in vitro*, we observed the morphology of cell seizure activity in culture medium containing Y27632 or exoenzyme C3. MHCC97H cells were seeded onto Matrigel, and the formation of capillary-like structures was monitored and photographed after 8- and 24-h incubation (Figure. 2A and B). As shown in (Figure. 2B, left panel and C), Y27632 at concentrations of 10 µM and 50 µM dose-dependently reduced the formation of tubule-like structures (**P<0.01 and ***P<0.001, *vs.* the control). Compared with the control, the formation of the tubular network was reduced almost by 80% at 50 µM of the compound, which provided the first evidence addressing the importance of this pathway in channel formation. Strikingly, there was no prominent dose-dependent destruction in the preformed VM structures with exoenzyme C3 treatment at the indicated concentrations in MHCC97H cells (*P*>0.05, Figure. 2B, right panel and D). These data suggest that RhoA may not be essential for the VM process. Altogether, Y27632 treatment was sufficient to abolish tube structures through blocking ROCK activity, indicating the potential role of ROCK as a mediator of VM.

### ROCK expression is consistent with the process of VM formation

**Figure 3:**
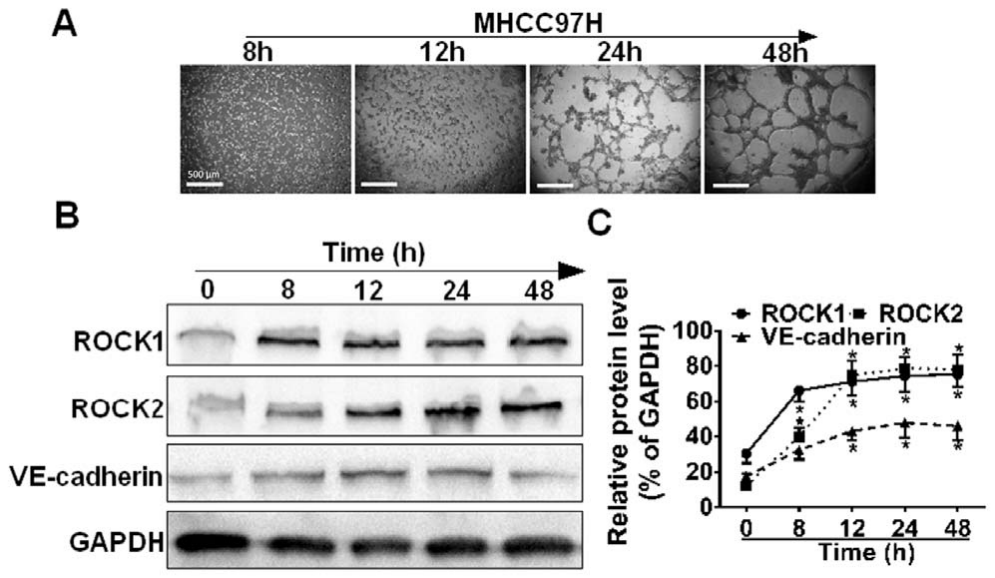
ROCK expression is consistent with the process of VM formation. (**A**) Formation of tubular network structures on Matrigel over 48 h. MHCC97H cells were subjected to Matrigel and photographed at 8, 12, 24 and 48 h, scale bars represent 500 µm. (**B**) Cell lysates from MHCC97H cells were seeded onto matrigel at the indicated time points and western blotted. (**C**) Relative protein level of each condition was quantitated using Image J and represented as line chart corresponding to pixels detected, (n = 3).

It was found that MHCC97H cells began aggregating, adhering and gradually forming characteristic tubular structures within 8 h after plating on Matrigel, and this process was basically completed within 24 h (Figure. 3A). To see whether the up-regulation of RhoA/ROCK contributed to VM formation, western blot analysis of whole cell lysates from these experimental samples was conducted to assess the expression of ROCK and VM-associated marker (VE-cadherin). As shown in (Figure. 3B and C), the expression level of ROCK varied in a time-dependent manner similar to the case with VE-cadherin. In addition, the levels of both ROCK1 and ROCK2 began rising significantly at 8 h (*P<0.05, *vs.* 0 h), and peaked at 24 h, which is consistent with the process of VM formation. These observations indicate that RhoA/ROCK signaling pathway was positively correlated with the potential of VM formation, which is consistent with the correlation that we observed between RhoA/ROCK signaling pathway and VM formation.

### Blockage of ROCK inhibits VM formation by reducing EMT

**Figure 4:**
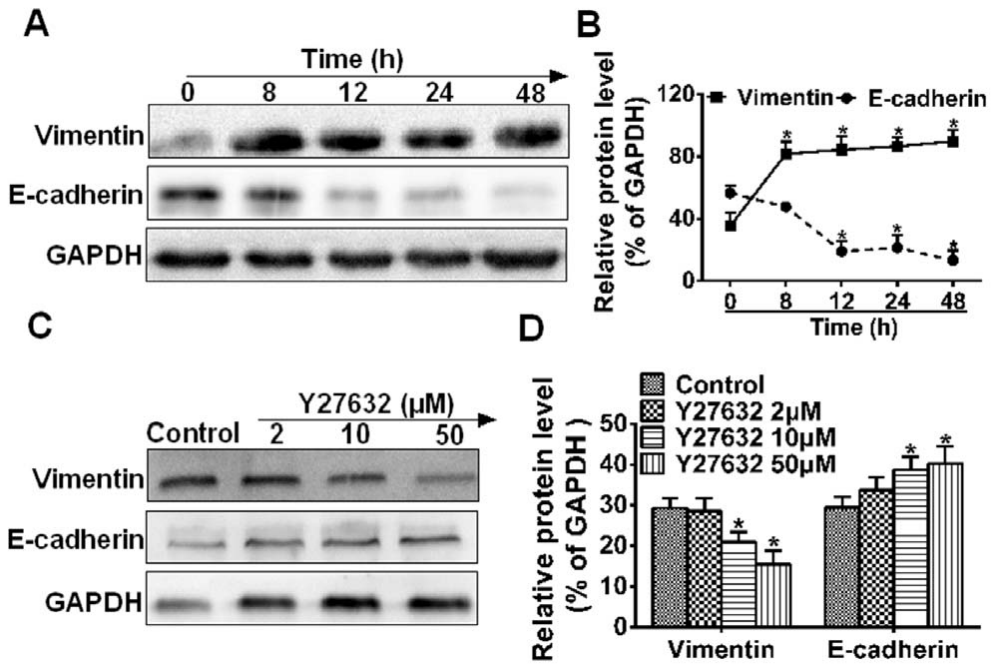
The effect of ROCK inhibition on the acquisition of an EMT phenotype. Western blot analysis showed that MHCC97H cells subjected to Matrigel with culture medium treated (**C**) or not treated (**A**) with Y27632 for 48 h regulated Vimentin and E-cadherin expression. GAPDH was used as a loading control. Quantification of EMT markers is shown in (**B**) and (**D**). The data are reported as mean ± S.E. (*P<0.05 compared to 0 h or the control, n = 3).

Previous studies [15] suggested that EMT was involved in the VM formation in HCC. To determine the correlation between ROCK and EMT in VM formation, we decided to analyze the effect of Y27632 on EMT markers (Vimentin and E-cadherin). As shown in (Figure. 4A and B), MHCC97H significantly increased the expression of mesenchymal marker (Vimentin) and decreased the expression of epithelial marker (E-cadherin) in a time-dependent manner during VM formation, compared with 0 h (*P<0.05). In contrast, the process was reversed upon Y27632 mediation at 10 and 50 µM (Figure. 4C and D). These data suggest that blockage of ROCK vanished VM formation in MHCC97H by reducing the occurrence of EMT.

### Y27632 induces a robust alteration in protein and gene expression profiles related to VM

**Figure 5:**
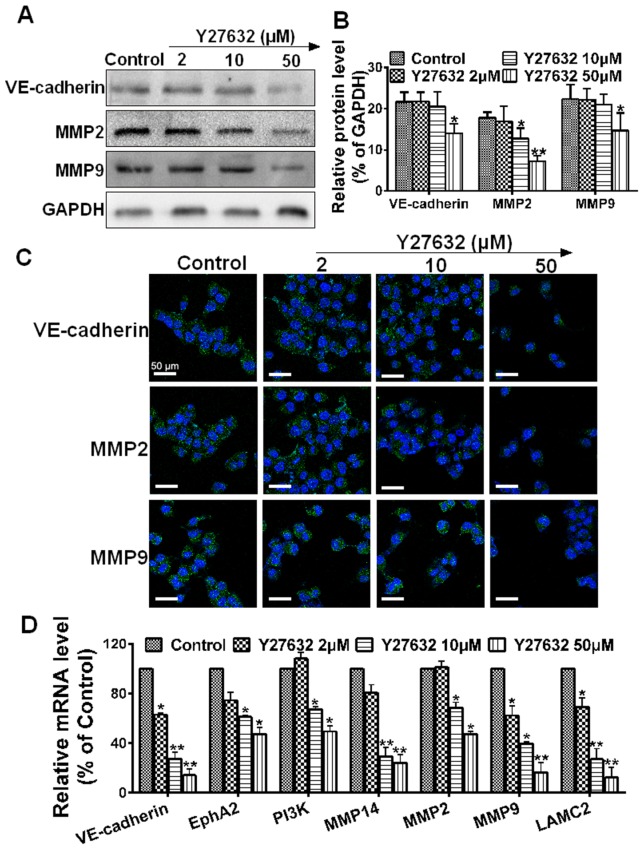
The effect of ROCK inhibition on the expression of VM-key factors. (**A**) Western blot assay was performed to evaluate the effect of ROCK inhibition on VM markers. (**B**) Relative protein level was quantitated using Image J (*P<0.05 and **P<0.01, ROCK inhibitor groups *vs.* the control, using GAPDH as an internal control for protein loading. (**C**) Representative confocal images (n = 3, five pictures per condition) of control and Y27632 (2, 10 or 50 µM) with 24 h incubation, after immunostaining for VE-cadherin, MMP2 or MMP9 (green) and the nuclear dye DAPI (blue), scale bars represent 50 µm. (**D**) Real-time qPCR analysis was used to determine changes in gene expression. Relative Y27632-induced change in gene expression compared with GAPDH is expressed as fold change calculated by 2^-ΔΔCp^ method. Data are expressed as mean ± S.E. from three independent experiments, with significant differences from control shown as *P<0.05 and **P<0.01.

Signaling cascades involved in vasculogenic mimicry can be categorized into pathways associated with vascular, embryonic and/or stem cells, and hypoxia signaling [7], while vascular signaling pathways are considered critical for vasculogenic events [17]. To further characterize the RhoA/ROCK signaling pathway mediating VM activation, western blot of whole cell lysates from these experimental samples was performed after addition of Y27632. As shown in (Figure. 5A and B), Y27632 suppressed the expression of protein in a dose-dependent manner. More specifically, VE-cadherin, MMP2 and MMP9 were down-regulated markedly after treatment with Y27632 (50 µM) as compared with the controls (*P<0.05), while the expressions of VE-cadherin and MMP9 were not affected significantly in the other two groups. The immunofluorescence analysis showed that no differences in VE-cadherin and MMP9 were observed between the control group and the Y27632 (2 and 10 µM) groups after 24 hours of incubation (Figure 5C), which is consistent with the results detected by western blot. To further deliberate the mechanism underlying the Y27632-mediated anti-vasculogenic effect, we evaluated six important tumor vasculogenic genes, using the primers shown in **Table 1**. Analyses of VE-cadherin, EphA2, PI3K, MMP14, MMP2 and MMP9 by real-time qPCR (Figure. 5D) showed that the Y27632 (50 µM) significantly inhibited mRNA levels, as compared with the controls (*P<0.05). Taken together, these results demonstrate that Y27632 had the potential to exogenously affect VM activity of MHCC97H cells in a consistent manner, strongly implying that the RhoA/ROCK pathway was involved in VM development.

**Table 1:** Sequences of primers.

Gene	Forward Primer (5′-3′)	Reverse Primer (5′-3′)	Size
VE-cadherin	CATTTGTCGTGCCTGAAGAC	ATGGTGAAAGCGTCCTGGTA	133
EphA2	CCCGGAGGACGTTTACTTCT	GGATGGATGGATCTCGGTAG	124
PI3K	CGTTTCTGCTTTGGGACAAC	CCTGATGATGGTCGTGGAG	100
MMP14	GAGGGTCTTCGTTGCTCAGT	GGGTTTCTTCTGCCCACTT	120
LAMC2	ACATTCCTGCCTCAGACCAC	TCCCTTGTCAGTTGCTCCAT	121
MMP2	TATGGCTTCTGCCCTGAGAC	CACACCACATCTTTCCGTCA	142
MMP9	CAGTCCACCCTTGTGCTCTT	ACTCTCCACGCATCTCTGC	118

All the sequences were based on the published data on the National Center for Biotechnology Information followed by the accession number.

### ROCK inhibitors suppress HCC cell motility

**Figure 6:**
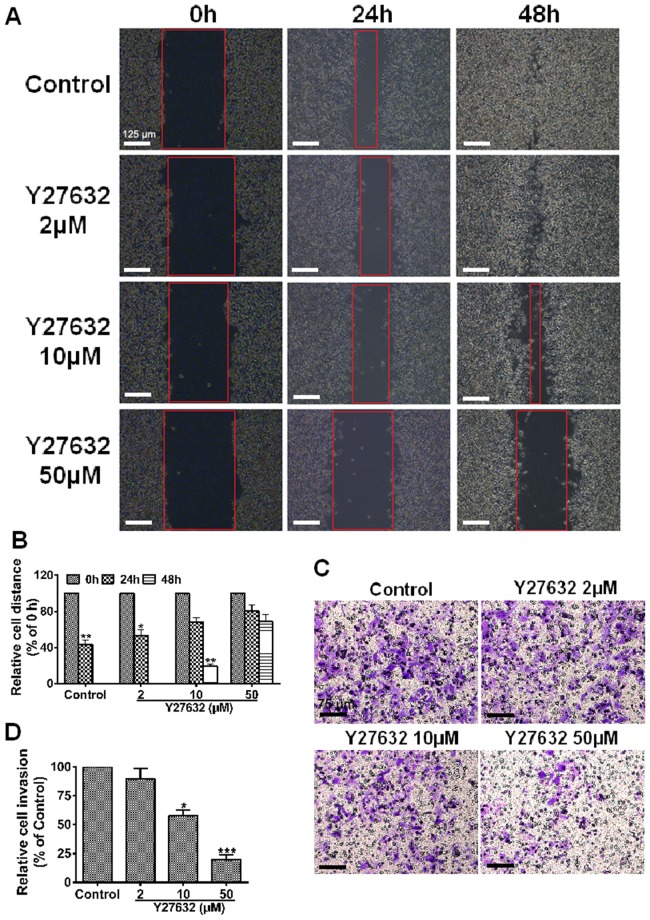
ROCK inhibition decreases the motility of MHCC97H cells. (**A**) Y27632 inhibition delayed wound healing. Representative images of scratch wounds formed at 0, 24 and 48 h after wounding. Original magnification was 100×, scale bars represent 125 µm. (**C**) Y27632 inhibition decreased invasiveness. Invaded cells attached at the lower surface of the filters were stained with crystal violet. Original magnification was 200×, scale bars represent 75 µm. (**B**) and (**D**) Data were normalized to untreated cells and the relative migration or invasion is expressed as mean ± S.E. of triplicate experiments. Fewer cells migrated or invaded 48 h after incubation with Y27632 (50 µM), compared with the control cells, *P<0.05, **P<0.01 and ***P<0.001.

VM is believed to be associated with cell migration and invasion. To further determine the active role of ROCK in the development of a vascular phenotype of MHCC97H, MHCC97H cells treated with Y27632 (2, 10 and 50 µM) were subjected to scratch wound assays and invasion assay. Cell migration toward the wounded area was observed (Figure. 6A). No treatment of MHCC97H led to nearly complete wound closure, while addition of Y27632 (50 µM) to cells led to a significant blockage of migration (*P*>0.05 *vs.* 0 h, Figure. 6B). These results indicate that Y27632 treated cultures exhibited a failure to migrate. In addition, cell invasion decreased by about 45% and 75% after blockage with in 10 µM and 50 µM Y27632 respectively, as compared with control cells (*P<0.05 and ***P<0.001, Figure. 6C and D). These findings indicate that the inhibition on cell motility by Y27632 was significant and concentration dependent.

### Y27632 treatment did not affect cell apoptosis and viability

**Figure 7:**
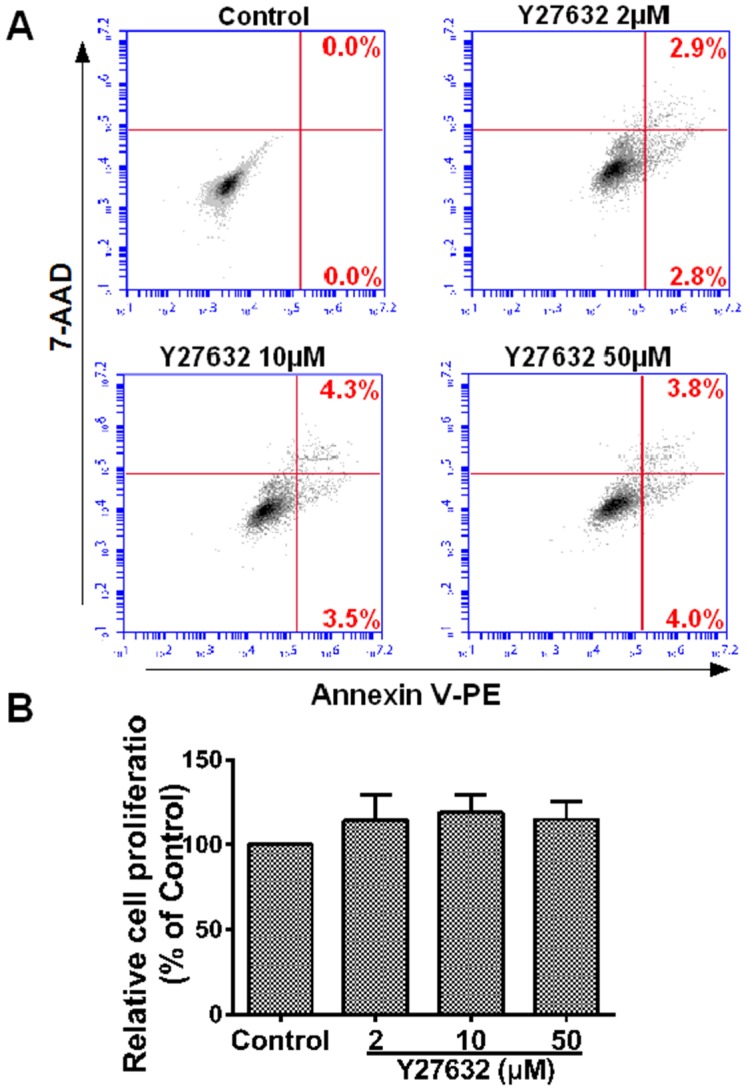
Y27632 treatment did not affect cell apoptosis and viability. (**A**) The concentrations of Y27632 (up to 50 µM) did not induce apoptosis of MHCC97H cells according to flow cytometric analysis. (**B**) Y27632 treatment did not affect cell viability. There was no statistically significant difference from three independent experiments (*P*>0.05, n = 5 in each group).

To eliminate any misinterpretation due to cell apoptosis or death caused by toxicity of the compound, we performed cell apoptosis and viability assessment on HCC cells. Flow cytometric analysis showed Y27632 up to 50 µM was found to be little cytotoxic to MHCC97H cells (*P*>0.05, *vs.* the control, Figure. 7A). Cell proliferation assay showed that Y27632 did not affect cell growth (*P*>0.05 *vs.* the control, Figure. 7B), confirming that inhibition of ROCK only affected HCC cell migration.

## Discussion

Invasion of malignant tumor cells into neighboring tissues and formation of metastasis are crucially dependent on the migratory properties of the cancer cell. Most *in vitro* studies investigated Rho GTPase function in mesenchymal migration using a two-dimensional system: RhoA, B and C to stimulate the formation of stress fibers and focal adhesions, actomyosin-mediated cell contraction, and rear detachment [19]. In addition, Rho GTPases control the directionality of cell migration. In the present study, we examined the expression levels of RhoA, ROCK1 and ROCK2 in HCC cell lines and revealed a positive correlation between high RhoA/ROCK expression and high invasive potential. VM is defined as a fluid-conducting channel formed by highly invasive and genetically dysregulated melanoma cells [20] and has become a prognostic factor for poor clinical outcomes [8], [21]. Our assay on the formation of tubular network structures demonstrated that higher levels of RhoA/ROCK were more prone to form VM networks, compared with RhoA/ROCK low-expressing cells, suggesting that the RhoA/ROCK signaling pathway is positively correlated with the potential of VM formation in HCC cells. In addition, the expression level of ROCK altered in a time-dependent manner as was the case with the formation of VM, confirming the contribution of RhoA/ROCK to VM formation.

Tumor cell invasion and metastasis require a complex cascade of events including changes in the cytoskeleton. Accumulating evidence indicates that the RhoA and downstream target ROCK plays an important role in oncogenesis. The interaction of GTP-bound RhoA to Rho-binding domain (RBD) of ROCK increases ROCK activity through de-repression of the carboxyl-terminal RBD-pleckstrin homology (PH) domains on the amino-terminal kinase domain, leading to an active “open” kinase conformation [22]. It was reported that gastric cancer cell invasion could be induced via activation of the RhoA/ROCK pathway by IL-6 [23], and RhoA and RhoC siRNA gene therapy mediated by adenovirus has been suggested as useful for inhibiting the growth and invasion of human gastric carcinoma via the PI3K/Akt pathway [24]. However, our finding that exoenzyme C3 did not destruct VM structure formation suggests that RhoA is not essential for the VM formation process, probably because ROCK is an evolutionarily conserved downstream effector of RhoA, RhoB, RhoC and RhoE, all of which are potential activators [25]. Therefore, blockage of each GTPase above directly binds to ROCK and inhibits its kinase activity. Differently, RhoA activates ROCK1 through interaction with the carboxy-terminal site. RhoE and RhoA are unable to bind ROCK1 simultaneously [26], and RhoE phosphorylation events seem to antagonize RhoA-induced stress fiber assembly [27]. Interestingly, ROCK could also be activated independently of RhoA through amino-terminal transphosphorylation caused by protein oligomerization. Other small GTP-binding proteins such as Gem and Rad specifically regulate either ROCK1- and ROCK2-mediated cell rounding and neurite retraction [28]. In addition, ROCK can be auto-phosphorylated, suggesting that the function of ROCK may be, in part, dependent on auto-regulation [29]. However, which mechanism induces RhoA as not essential for the VM formation process needs to be determined by further investigation.

The ROCK family includes ROCK1 and ROCK2 that share 65% overall identity and 92% identity in the kinase domain. Both kinases contain a catalytic kinase domain at the N-terminus, followed by a central coiled-coil domain, including a RBD and a C-terminal PH domain, with an internal cysteine-rich domain. ROCK promotes actin filament stabilization and the generation of actin-myosin contractility by phosphorylating numerous downstream target proteins, including the myosin binding subunit of myosin light chain (MLC) phosphatase (MYPT1), MLC2, LIM kinases, ezrin/radixin/moesin and adducin. Y27632 or fasudil, inhibitors of ROCK, cause loss of actin stress fibers and focal adhesion complexes [30]. This is predominantly due to the phosphorylation and inhibition of MLCP by ROCK, which increases MLC phosphorylation and cellular contraction by facilitating interaction of myosin with F-actin [22]. Thus, ROCK regulates cell polarity and migration through cellular contractions, protrusions, and focal adhesions. It was found in the present study that Y27632 reduced the formation of tubule-like structures in a dose-dependent manner, indicating the potential role of ROCK as a mediator of VM. However, further studies are required to reveal which ROCK isoform dominates the VM activity.

Epithelial cells can convert into mesenchymal cells through a process known as EMT. EMT refers to a series of events in which epithelial cells lose many of their epithelial characteristics and take on properties typical of mesenchymal cells that require complex changes in cell architecture and behavior. The mechanism between the VM channels in highly aggressive human tumor cells and the EMT process may share the same process or similar relevance. These findings in (Figure. 4) suggest that VM formation is correlated with the EMT-marker and ROCK inhibition reverses the process of EMT, supporting the idea that VM formation may be part of EMT [31].

The key signaling pathway of VM formation is described as colocalization of VE-cadherin and activation of PI3K by EphA2, which subsequently induce the expression and activation of MMP14, which subsequently triggers MMP2 activation. Altogether, MMP14 and MMP2 promote the cleavage of LAMC2 to the pro-migratory γ2′ and γ2× fragments. It was suggested that up-regulation of any molecule in the above tumor microenvironment could increase migration and invasion, and may ultimately result in VM. However, what initially triggers this pathway to become activated remains unknown. In recent years, many more studies linking various angiogenesis-promoting factors to VM have been published. Vascular endothelial growth factor-A (VEGF-A) promoted the up-regulation of VM-associated genes for VE-cadherin, EphA2, MMP2 and MMP9 [32]. In contrast, our investigation showed that VM-associated genes was down-regulated, suggesting that ROCK inhibition could attenuate the characteristics associated with tumor cell plasticity essential for VM. Therefore, we conclude that VEGF-A and ROCK may have the same effect on VM formation. Similar findings in other studies [33] demonstrated that selective blockage of ROCK with Y27632 dose-dependently inhibited VEGF-induced venular hyperpermeability. We therefore hypothesize that VE-cadherin up-regulation through specific induction by ROCK activates the subsequent signaling cascade, similar to VEGF-A. Our future study will address the potential significance of this relationship. The recent progress in Rho/ROCK research continuously supports the therapeutic importance of the ROCK pathway in migration. MMPs have been implicated in a variety of mechanisms that promote tumor progression [34]. In particular, MMP2 and MMP9 are key mediators of invasion, metastasis and tumor angiogenesis [35]. Our western blot and real-time qPCR assays showed that both MMP2 and MMP9 were down-regulated. This finding could serve as an explanation why Y27632 not only suppressed cell migration but cell invasion that involves the extracellular matrix barrier (Figure. 6). In contrast, other studies reported contradictory findings. One study [2] reported that MMP9 but not MMP2 was correlated with VM formation in HCC, and another study [36] reported that Y27632 promoted human trabecular meshwork cells in adhesion, contraction and motility. However, there is also growing evidence to support the importance of Rho/ROCK in multiple aspects of migration activities [37]–[39]. Given the volume of the literature supporting a role for Rho/ROCK in controlling cell movement, it is not surprising to find that inhibition of ROCK blocks MHCC97H migration and invasion as demonstrated in our study.

In the current study, we demonstrated that the expression level of RhoA/ROCK was different in HCC cells, and that higher active RhoA/ROCK expression was prone to form VM channels. In addition, ROCK, but not RhoA, played a crucial role in the development and progression of cancer VM formation *in vitro* in MHCC97H cell line. Moreover, EMT was correlated with ROCK-induced VM formation. In brief, our results suggest that the RhoA/ROCK pathway is involved in numerous aspects of the VM process, and ROCK may prove to be a therapeutic target for VM in HCC.

## Materials and Methods

### Chemical and antibodies

Chemical and antibodies used in this study included Matrigel (BD Biosciences); cell culture media (RPMI 1640, DMEM and MEM); fetal bovine serum and antibiotics (Gibco); exoenzyme C3, Y27632 and other chemicals (Sigma-Aldrich); anti-VE-cadherin antibody (Cell Signaling); PE Annexin V Apoptosis Detection Kit (BD Pharmingen). The rest were purchased from Abcam Inc. (Cambridge).

### Cell culture

HUVEC, human hepatocyte cell line LO2 and MHCC97H cells were obtained from the Liver Cancer Institute, Zhongshan Hospital, Fudan University (Shanghai, China). SMMC7703, SMMC7721, HepG2 and Hep3B were from the cell bank of the Chinese Academy of Sciences. All the cell lines were cultured separately in RPMI 1640 (HUVEC, SMMC7703, SMMC7721 and LO2), MEM (HepG2 and Hep3B) and DMEM (MHCC97H), supplemented with 10% FBS and antibiotics. All cultures were maintained at 37°C in a humidified atmosphere of 5% CO_2_.

### Matrigel tube formation assay

The Matrigel tube formation assay is a technique used to determine *in vitro* VM capacity of cancer cells. Briefly, each well of a 24-well plate was coated with 100 µl Matrigel (10 mg/ml) and allowed to solidify at 37°C for 1 h. The cell suspension in culture medium (4×10^4^/200 µl) was seeded into the Matrigel-coated 24-well plate and incubated at 37°C in a humidified atmosphere of 5% CO2. The effect of RhoA/ROCK inhibition on tube formation was studied by adding culture medium containing exoenzyme C3 or Y27632 into the wells immediately after cell seeding to a final concentration of 200, 800 or 1600 ng (exoenzyme C3) or 2, 10 or 50 µM (Y27632).

### Western blot analysis

Total protein was extracted and electrophoresed using SDS-PAGE. Proteins on the gel were transferred onto PVDF membrane (Merck-Millipore), followed by blocking with 5% skimmed milk dissolved in TBS containing 1% Tween-20 (TBST) for 1 h at room temperature. The membrane was incubated with primary antibody at 4°C overnight, washed with 1% TBST three times, and incubated in alkaline phosphatase-conjugated secondary antibody for 1 h at room temperature. After washing, the chemiluminescence signal was imaged using a ChemiDoc XRS (Bio-Rad) and quantitated using Image J.

### cDNA generation and real-time qPCR

Cells were treated with 2, 10 or 50 µM of Y27632 for 16 h. Total RNA was extracted from cells using Trizol (Invitrogen) and verified by electrophoresis. The mRNA was reverse-transcribed into cDNA with a reverse-transcription kit (Thermo Fisher Scientific). Human GAPDH Endogenous Reference Genes Primers (Order NO.: PHS04) and Primer sequences listed in **Table 1** used for PCR were from Sangon Biotech. SYBR Green I (Amersco) was added into the reaction mixture. Quantitative real-time PCR was performed on an MJ Opticon 2 thermal cycler (MJ Research Inc.) following the manufacturer's instruction.

### Immunostaining

After fixation for 20 min in 4% paraformaldehyde at 37°C, cells were permeabilized for 15 min in 0.5% Triton-X and subsequently blocked in 5% goat serum for 1 h. After incubation with the appropriate primary (overnight incubation at 4°C) and secondary (2 h at 37°C) antibodies, the cells were imaged using a confocal laser scanning microscope (Leica TCS SP8).

### Cell migration assay

Cell migration was evaluated using an *in vitro* wound healing assay. In brief, MHCC97H cells containing Y27632 at a final concentration of 2, 10 or 50 µM were seeded to a six-well plate and incubated for 8 h to allow the formation of a cell monolayer. Cells were scratched with the tip of a 200 µl pipette and then incubated at 37°C in a humidified atmosphere of 5% CO_2_ for 48 h. To observe the scratch healing, each well was observed at 0, 24 and 48 h after scratching.

### Matrigel invasion assay

Invasion was assayed in Transwell cell culture chambers (Corning Costar) attached with a membrane filter (8.0 µm pore size; Nucleopore) as previously described with some modifications [40]. Briefly, the inserts in the membrane filter were coated with Matrigel on the upper surface. MHCC97H cells were adjusted to a density of 1×10^6^ cells/ml in serum-free DMEM high glucose culture medium. Cell suspension (200 µl) was seeded into the upper surface, while the lower chambers were filled with 500 µl DMEM medium with 20% FBS. After culture at 37°C in a humidified atmosphere of 5% CO_2_ for 48 h, the cells on the upper surface of the filter were removed by wiping with a cotton swab and the number of cells that migrated through the Matrigel to the lower chamber was determined. Assays were performed in duplicate wells.

### Cell apoptosis and viability assessment

Apoptosis assay of MHCC97H cells exposed to Y27632 (2, 10 and 50 µM) for 24 h were examined by flow cytometry (BD Accuri C6). MHCC97H cells were stained with PE Annexin V and 7-Amino-Actinomycin (7-AAD) following the manufacturer's instructions to detect early apoptotic cells (PE Annexin V^+^/7-AAD^-^ events) and late apoptotic cells (PE Annexin V^+^/7-AAD^+^ events). Additionally, MHCC97H cells were trypsinized and seeded at 1×10^4^ cells/well in 96 well plates. After 24 h, various concentrations of Y27632 were added, followed by incubation for another 48 h. Then, 10 µl Cell Counting Kit-8 (CCK8, Dojindo) solution was added to each well. Plates were incubated for additional 2 h. Absorbance readings at 490 nm were obtained using a spectrophotometer (Thermo Varioskan).

### Statistical analysis

The data were expressed as mean ± standard errors (S.E.) and examined for their statistical significance of difference with ANOVA and the Student's *t*-test. *P*-values of less than 0.05 were considered statistically significant.
